# 2018 Korean Society of Hypertension guidelines for the management of hypertension: part I-epidemiology of hypertension

**DOI:** 10.1186/s40885-019-0121-0

**Published:** 2019-08-01

**Authors:** Hyeon Chang Kim, Sang-Hyun Ihm, Gheun-Ho Kim, Ju Han Kim, Kwang-il Kim, Hae-Young Lee, Jang Hoon Lee, Jong-Moo Park, Sungha Park, Wook Bum Pyun, Jinho Shin, Shung Chull Chae

**Affiliations:** 1 Department of Preventive Medicine, Yonsei University College of Medicine, Seoul, South Korea; 2 Department of Internal Medicine, College of Medicine, The Catholic University of Korea, Seoul, South Korea; 3 Department of Internal Medicine, Hanyang University College of Medicine, Seoul, South Korea; 4 Department of Internal Medicine, School of Medicine, Chonnam University, GwangJu, South Korea; 5 Department of Internal Medicine, Seoul National University Bundang Hospital, Seoul National University College of Medicine, Seongnam, South Korea; 6 Department of Internal Medicine, Seoul National University College of Medicine, Seoul, South Korea; 7 Division of Cardiology, Department of Internal Medicine, Kyungpook National University School of Medicine, 130 Dongdeok-ro, Jung-gu, Daegu, 700-721 South Korea; 8 Department of Neurology, Eulji General Hospital, Eulji University College of Medicine, Seoul, South Korea; 9 Department of Internal Medicine, Yonsei University College of Medicine, Seoul, South Korea; 10 Department of Internal Medicine, School of Medicine, Ewha Womans University, Seoul, South Korea

**Keywords:** Hypertension, Guideline, Epidemiology, Prevalence, Awareness, Treatment, Control, White coat hypertension, Masked hypertension

## Abstract

The Korean Society of Hypertension guideline defines hypertension as systolic blood pressure ≥ 140 mmHg or diastolic blood pressure ≥ 90 mmHg, where the effectiveness of pharmacological treatment has been established. It is confirmed that higher blood pressure levels are associated with increased risk of cardiovascular disease and mortality also in the Korean population. About one third of Korean adults aged 30 years or older are estimated to have hypertension, and the prevalence of hypertension gradually increases as the age increases. The awareness, treatment, and control rates of hypertension are generally improving in Korea, but more efforts are required to increase awareness and treatment among younger patients with hypertension and to improve lifestyle modification compliance at all ages. More studies are required to determine the magnitude and impact of white coat hypertension and masked hypertension in the Korean population.

## Classification of blood pressure and hypertension

This guideline defines ‘hypertension (HTN)’ as a blood pressure (BP) above the threshold value for which the effectiveness of pharmacological treatment has been proved by randomized clinical trials and also classifies BP values lower than the threshold value based on cardiovascular (CV) risk for using lifestyle modifications to lower BP and prevent HTN. Therefore, based on the available evidence from clinical trials, HTN is defined as systolic blood pressure (SBP) ≥140 mmHg or diastolic blood pressure (DBP) ≥90 mmHg. HTN is further classified into grade 1 and grade 2 HTN depending on the higher SBP or DBP value. ‘Normal BP’ is defined only as both SBP below 120 mmHg and DBP below 80 mmHg. Normal BP defines the optimal condition with the lowest CV risk and is used as a reference when assessing the risks of higher BP [1]. When an individual’s SBP is 120 to 129 mmHg and the DBP is lower than 80 mmHg, the individual is considered to have ‘elevated BP’. Individuals with a SBP of 130 to 139 mmHg or a DBP of 80 to 89 mmHg are considered to have ‘prehypertension’. When an individual’s SBP is greater than or equal to 140 mmHg but DBP is below 90 mmHg, the patient is considered to have ‘isolated systolic HTN’ (Table 1).

**Table 1:** Classification of blood pressure and hypertension

Category		Systolic blood pressure (mmHg)		Diastolic blood pressure (mmHg)
Normal blood pressure^a^		< 120	And	< 80
Elevated blood pressure		120–129	And	< 80
Prehypertension		130–139	Or	80–89
Hypertension	Grade 1	140–159	Or	90–99
	Grade 2	≥160	Or	≥100
Isolated systolic hypertension		≥140	And	< 90

^a^Blood pressure with minimal risk for cardiovascular events

## High blood pressure and risk of cardiovascular disease

**Fig. 1:**
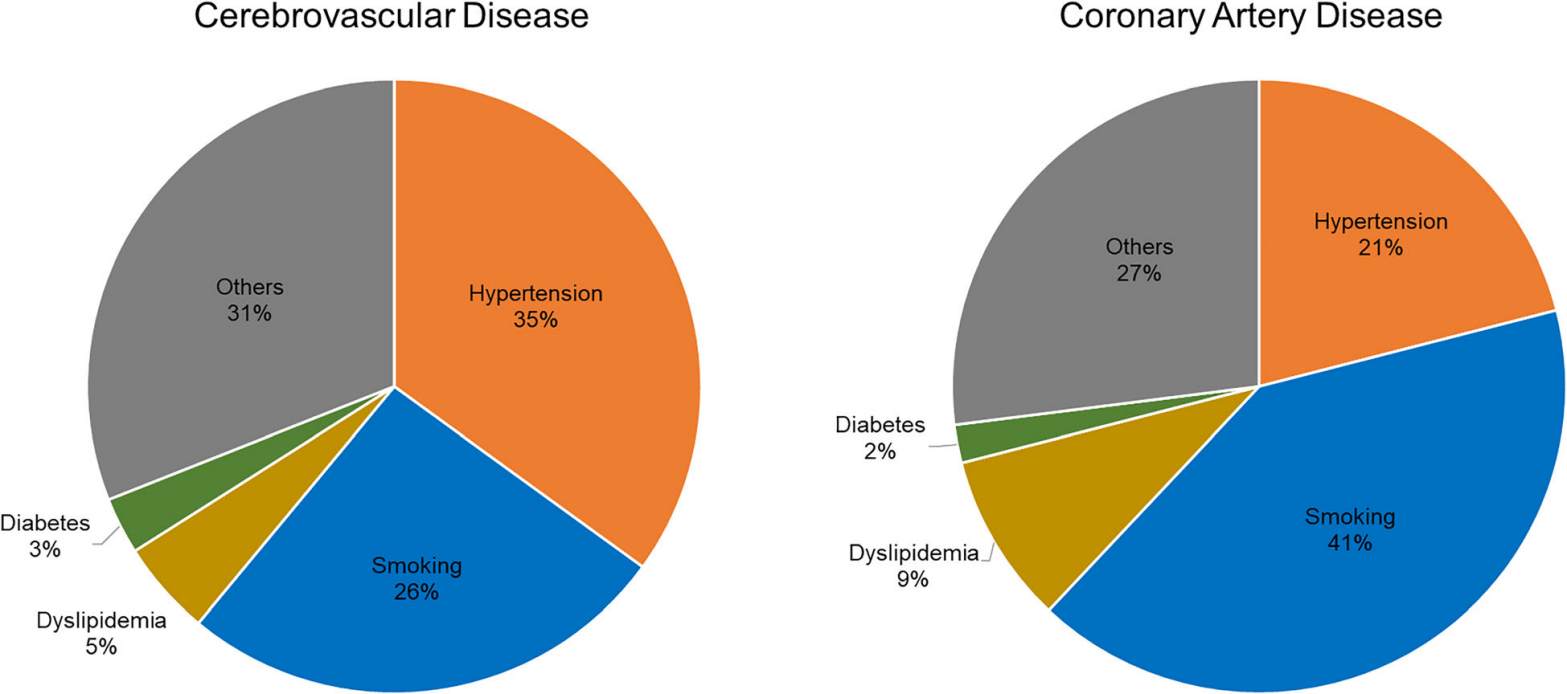
Attributable risks of major cardiovascular risk factors for cerebrovascular disease and coronary artery disease in Korean men (Source: Korean Medical Insurance Corporation study)

It is well known that higher BP levels are associated with increased risk of cardiovascular disease (CVD) and mortality. In the Korean Medical Insurance Corporation (KMIC) study, individuals with SBP/DBP greater than or equal to 140/90 mmHg showed 2.6-fold higher risk of cerebrovascular disease and coronary artery diseases (CAD), compared to those with SBP/DBP below than 130/85 mmHg [2, 3]. In a case-control study within the KMIC cohort, higher BP was the most contributable risk factor to cerebrovascular disease and was associated with the risk of CAD [3, 4]. Both Western and Asian studies have shown that individuals with elevated BP or prehypertension tend to have a worse lifestyle compared to those with normal BP. In addition, the probability of progressing to HTN and the risk for a CV event were higher in the elevated BP or prehypertension groups than those in the normal BP group [5–7]. In a large-scale pooled cohort study of Asians, including many Koreans, HTN was an important risk factor for the development of stroke and ischemic heart disease [8]. The study also predicted that stroke prevention by BP control would be much greater in Asian populations, since the risk of stroke was reduced by 41% in Asians and 30% in Caucasians when SBP was 10 mmHg lower [1]. As shown in Fig. 1, the attributable risks to HTN for cerebrovascular disease and CAD in Korean men were 35 and 21%, respectively. In addition, for each 20 mmHg increase in SBP, the relative risks of ischemic stroke, intracerebral hemorrhage, and subarachnoid hemorrhage were 1.79, 2.48, and 1.65, respectively, in men and 1.64, 3.15, and 2.29, respectively, in women [9]. Therefore, the contribution of HTN to stroke and CAD has been well established in Korean studies, and stroke is strongly associated with HTN.

## Prevalence of hypertension and distribution of blood pressure

### Prevalence of hypertension

Since 1998, the Korean National Health and Nutrition Examination Survey (KNHANES) has measured resting upper arm BP for a representative sample of the Korean population and has defined HTN as SBP ≥140 mmHg, DBP ≥90 mmHg or taking antihypertensive medications. The age-standardized prevalence of HTN is approximately 30% among adults aged 30 years or older.

### Changes in the prevalence of hypertension

As shown in Table 2, the age-adjusted prevalence of HTN from the KNHANES decreased from 29.8% in 1998 to 25.9% in the years 2007–2009 then increased again up to 29.1% (men 35.0%, women 22.9%) in 2016 [10, 11]. In addition, 25.9% of individuals (men 30.8%, women 20.8%) have elevated BP or prehypertension. Thus, 55% of Koreans aged 30 years or older have higher than normal BP. This percentage increases to 65.2% (men 61.7%, women 67.7%) among individuals aged 65 years or older, and increases further in women compared to men.

**Table 2:** Trends in the prevalence of hypertension in the population aged ≥30 years (Source: KNHANES)^a^

Category	1998	2001	2005	2007–09	2010–12	2013–15	2016
All	29.8%	28.5%	28.0%	25.9%	28.0%	26.8%	29.1%
Men	32.4%	33.2%	31.5%	28.8%	31.4%	31.6%	35.0%
Women	26.8%	25.3%	23.8%	22.7%	24.2%	22.0%	22.9%

^a^Age-standardized values were calculated using the age structures of the 2005 Korean population

### Age and sex differences in blood pressure

**Fig. 2:**
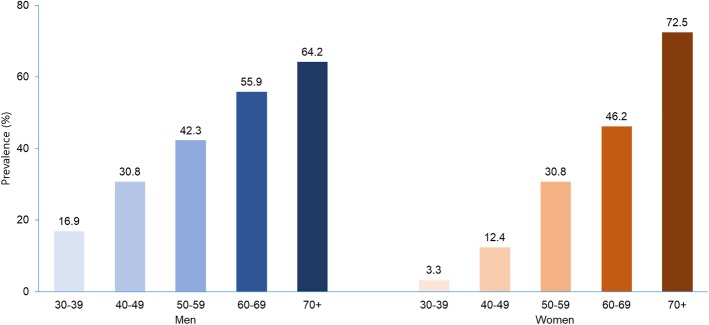
Prevalence of hypertension by sex and age (Source: 2016 KNHANES)

As the age increases, BP increases gradually, and the prevalence of HTN exceeds 50% at age 60 and over (Fig. 2) [12]. In the KNHANES 2016, the prevalence of HTN was 50.9% among individuals aged 60–69 years and 69.2% among individuals aged 70 years or older. The prevalence was higher in men (55.9%) than in women (46.2%) until the age of 60, but lower in men (64.2%) than in women (72.2%) after age 70. In addition, SBP continues to increase with age, but DBP begins to decline after age 60, thus the pulse pressure, defined as the difference between SBP and DBP, increases in the elderly population.

### Salt intake and blood pressure

It is known that reducing salt intake can lower BP. The daily intake of salt in Koreans is estimated to be over 10 g, so it is important to reduce salt intake to prevent HTN [13]. According to an analysis of the 1998, 2001, and 2005 KNHANES data, younger patients with hypertension (aged less than 40 years) consumed more salt than older patients with hypertension, but there was no significant difference after adjusting for total energy intake [14–16]. However, an independent association between salt intake and BP was reported among individuals with metabolic syndrome [14–17]. In addition, the urinary sodium/creatinine ratio was more closely associated with the BP level than the salt intake [18]. Since the KNHANES evaluated salt intake with food questionnaires, the accuracy of individual salt intake is limited. Also, since the KNHANES is a cross-sectional study, it is not known whether diet has changed after the diagnosis of hypertension. More research is needed to understand the impact of salt intake on high blood pressure in the Korean population. Because the salt intake in KNHANES was estimated by using a food questionnaire, which has limited accuracy, and because it was a cross-sectional analysis including both patients with HTN who had not yet changed their diet as well as those who changed their eating habits after diagnosis, additional research is needed. According to studies in other countries, a reduction of salt intake is effective to lower BP regardless of the cross-sectional association between salt intake and BP [19].

### Metabolic syndrome and hypertension

Using the criteria for abdominal obesity as a waist circumference of greater than 90 cm for men and 80 cm for women, the prevalence of metabolic syndrome was 24.1% in the 1998–2005 KNHANES [20]. The prevalence of metabolic syndrome in Korean adults increased from 28.8 to 30.5% during the period from 2009 to 2013, and the increase in abdominal obesity was noticeable [21]. Among the components of the metabolic syndrome, high BP is the most common in men (40%), but low HDL-cholesterol level (59%) is the most common, followed by HTN (30%), in women [22]. Based on the data from the 2001 and 2005 KNHANES, the prevalence of metabolic syndrome was 53.3% in patients with HTN and 26.2% in people with elevated BP or prehypertension, both of which were significantly higher than the 24.1% prevalence in the general population [23]. Metabolic disorder is a determinant of progression from prehypertension to overt HTN [24, 25], thus, individuals with metabolic syndrome are the key targets for lifestyle modification.

## Management of hypertension

It is important to monitor the rates of awareness, treatment, and control of HTN, as these are the key indicators of the quality of HTN management. The awareness rate is defined as the proportion of the subjects who are aware of their physician-diagnosed HTN among all of the subjects with HTN. The treatment rate is defined as the proportion of the subjects taking antihypertensive drugs at the time of the survey among all of the subjects with HTN. The control rate is defined as the proportion of the subjects with SBP/DBP controlled below 140/90 mmHg among the subjects taking antihypertensive drugs or among all subjects with HTN.

The awareness, treatment, and control rates of hypertension in South Korea are generally improving. According to the data from KNHANES, the awareness rate among HTN patients aged 30 years or older increased from 25% in 1998 to 65% in 2016. The treatment rate also improved significantly from 22% in 1998 to 61% in 2016. During the same period, the control rate increased from 5 to 44% among all patients with HTN, and from 24 to 71% among patients taking antihypertensive drugs (Table 3). Although the prevalence of HTN in Korea has not changed much over the past 30 years, the average BP level has steadily decreased, especially among patients with HTN, which has led to the reduction in the mean BP of the entire population [10].

**Table 3:** Trends in awareness, treatment, and control rate of hypertension (Source: KNHANES)^a^

Parameter	1998	2001	2005	2007–09	2010–12	2013–15	2016
Awareness rate	25%	34%	57%	65%	63%	64%	65%
Treatment rate	22%	32%	50%	59%	58%	60%	61%
Control rate for all hypertension patients	5%	12%	27%	41%	40%	43%	44%
Control rate for treated hypertension patients	24%	38%	55%	69%	69%	72%	71%

^a^Age-standardized values were calculated using the age structures of the 2005 Korean population

The changes in these indicators suggest that the overall management of HTN has improved over the past few decades. However, the rates of awareness, treatment, and control are still low among younger patients with HTN, more specifically those in the 30s to 40s age groups. According to the 2016 KNHANES data, HTN awareness rates of individuals in their 30s and 40s are 17.1 and 42.4%, treatment rates are 15.0 and 35.3%, and the control rates are 8.0 and 26.0%, respectively. Although the control rates for all HTN patients is much lower in the younger age group than in the older group, but the control rate for treated HTN patients does not differ among age groups, this implies that early detection and treatment of HTN in younger individuals is important. According to the 2007 KNHANES data, the proportion of individuals actively performing three or more of the listed lifestyle modifications (weight control, regular exercise, moderation of alcohol intake, reduction of salt intake, and smoking cessation) was only 38.2% among patients with HTN aged 40 years or older. More efforts are required to improve lifestyle modification compliance.

## White coat hypertension and masked hypertension

‘White coat HTN’ is diagnosed when the patient’s BP in the doctor’s office is 140/90 mmHg or above but the daytime ambulatory BP or home BP is less than 135/85 mmHg. If the BP is high both in and out of the office, the patient is considered to have persistent HTN. According to the registry data for ambulatory BP monitoring (ABPM) in secondary or tertiary referral centers supported by the Korean Society of Hypertension (KorABP registry), 14.9% of 1916 subjects who underwent ABPM for the diagnosis of HTN were found to have white coat HTN. In a single-center study from a domestic tertiary hospital, white coat HTN occurred more frequently in women and in men with a low body mass index (BMI) [26]. According to the KorABP registry data, the proportion of subjects in whom daytime ambulatory BP was lower than 135/85 mmHg and office BP higher than 140/90 mmHg was 13.5% among all treated subjects and 21.3% among subjects with uncontrolled HTN by office BP [27].

‘Masked HTN’ is defined as the condition in which the office BP is less than 140/90 mmHg and the daytime ambulatory BP or home BP is 135/85 mmHg or higher. According to the KorABP registry data, masked HTN was observed in 17.6% of patients who underwent ABPM for the diagnosis of HTN. Masked HTN was also observed in 13.8% of patients taking antihypertensive medication and in 35.1% of subjects with controlled office BP [27].. In a Korean study performed in primary care clinics, the prevalence of masked HTN was 21.2%, and male sex, older age, and smoking were the independent predictors of masked HTN [28]. A study of patients at a tertiary care center reported that the number of pills and higher fasting blood glucose are associated with masked HTN [29].

There is no available data on white coat HTN and masked HTN in the general Korean population. According to data from other countries, patients with white coat HTN have a relatively good short-term clinical course. However, white coat HTN can develop into persistent HTN and increase the risk of CVD in the long-term, therefore, patients with white coat HTN should be monitored regularly [30]. In foreign studies, masked HTN has a similar [31, 32] or worse [33] prognosis, compared to persistent HTN. A Korean study reported more severe myocardial damage in patients with masked HTN than in those with white coat HTN [34].

## Data Availability

Not applicable.
